# Single-cell and single-nucleus transcriptomics of the dorsal root ganglion in neuropathic pain: cell-state remodeling and translational prospects

**DOI:** 10.1186/s12967-026-08732-8

**Published:** 2026-09-02

**Authors:** Xu-Ran Liao, Huan Zheng, Jiang-Yuan Luo, Bi-Xin Zheng

**Affiliations:** 1 Department of Pain Management, West China Hospital, Sichuan University, Chengdu, 610041 China; 2 Department of Anesthesiology, West China Hospital, Sichuan University, Chengdu, 610041 China; 3 Department of Anesthesiology, Sichuan Provincial People’s Hospital, Sichuan Academy of Medical Sciences, University of Electronic Science and Technology of China, Chengdu, 610072 China

**Keywords:** Neuropathic pain, Dorsal root ganglion, Single-cell RNA sequencing, Single-nucleus RNA sequencing, Satellite glial cells, Neuroimmune interactions, Translational medicine

## Abstract

Neuropathic pain remains a major unmet clinical challenge, as current therapies provide limited efficacy and poor tolerability. A major obstacle to developing effective analgesics is the pronounced cellular and molecular heterogeneity of the dorsal root ganglion (DRG), which integrates neuronal, glial, immune, and stromal responses to injury. Recent advances in single-cell RNA sequencing (scRNA-seq) and single-nucleus RNA sequencing (snRNA-seq) have enabled cell-type-resolved analyses of these responses and revealed disease-associated cellular states that are obscured by bulk tissue profiling, thereby offering new opportunities to redefine disease mechanisms and therapeutic priorities. This review synthesizes evidence from single-cell studies of peripheral nerve injury, diabetic painful neuropathy, and chemotherapy-induced neuropathy. Although these conditions share common biological processes, including neuronal state remodeling, disrupted glial homeostatic support, context-dependent immune responses, and extracellular matrix reorganization, they do not converge on a single conserved molecular program. Peripheral nerve injury is characterized by neuronal injury and repair states, reactive and metabolic glial remodeling, and neuroimmune recruitment and crosstalk. Diabetic neuropathy is associated with altered sensory coding, impaired satellite glial lipid support, and neurodegenerative remodeling. Chemotherapy-induced neuropathy exhibits substantial agent-specific heterogeneity, including subtype-selective neuronal vulnerability and metalloproteinase-dysregulated satellite glial states following paclitaxel treatment, as well as sex-associated fibrotic remodeling following bortezomib treatment. These cellular states may serve adaptive, maladaptive, or degenerative functions. However, because most available studies rely on single-time-point or repeated cross-sectional sampling, they cannot directly establish temporal progression or causal relationships. We further examine how human DRG atlases bridge experimental models and human disease by determining whether candidate molecular targets and cell states are conserved and by identifying their cellular localization. These datasets also highlight species-specific differences in neuronal organization and non-neuronal transcriptional programs that may limit the direct translation of findings from rodent models. Consequently, the primary translational value of DRG single-cell studies lies in elucidating disease mechanisms and prioritizing candidate therapeutic targets for further investigation. Advancing these discoveries toward clinical application will require complementary evidence from human genetics, human DRG transcriptomic datasets, functional validation studies, and clinical pharmacology. Collectively, these complementary approaches may facilitate the development of mechanism-based and cell-type-informed analgesic strategies.

## Introduction

Neuropathic pain is a chronic condition resulting from lesions or diseases affecting the somatosensory nervous system and clinically manifests as spontaneous pain, hyperalgesia, and mechanical allodynia. With etiologies ranging from diabetic peripheral neuropathy and chemotherapy-induced peripheral neuropathy to trigeminal neuralgia, neuropathic pain poses a substantial global health burden that is compounded by psychiatric comorbidities, such as anxiety and depression [1, 2]. Existing pharmacological management remains limited, with guideline-supported first-line treatments, including gabapentinoids, tricyclic antidepressants, and serotonin–noradrenaline reuptake inhibitors, providing incomplete relief for many patients, whereas opioid use is constrained by safety concerns and inconsistent long-term benefits [3–5]. This therapeutic inadequacy is largely attributable to the translational gap between preclinical models and human pathology, together with an incomplete understanding of the molecular heterogeneity underlying pain chronification.

The dorsal root ganglion (DRG), which harbors the somata of pseudounipolar primary sensory neurons, functions as a key first relay station for somatosensory transmission. Owing to its relatively permeable blood–nerve barrier, the DRG is uniquely vulnerable to systemic metabolic insults and neurotoxic agents [6]. Under pathological conditions, the DRG becomes an active site of pain-related remodeling. Changes in ion-channel expression, neuronal excitability, and ectopic discharge can increase afferent input to the spinal dorsal horn and contribute to central sensitization [7–9]. This remodeling extends beyond neurons to satellite glial cells, Schwann cells, immune cells, vascular cells, and stromal populations. Depending on the disease context and stage, these responses may contribute to tissue repair, sensory sensitization, or neurodegeneration [10–12].

Conventional bulk RNA sequencing averages gene expression across whole tissues and can therefore obscure cell-type-specific changes and signals from less abundant populations, including nociceptor subtypes and infiltrating immune cells [13–15]. The advent of single-cell RNA sequencing (scRNA-seq) and single-nucleus RNA sequencing (snRNA-seq) has enabled cell-type-resolved profiling of DRG heterogeneity [16]. Compared with scRNA-seq, snRNA-seq can improve the representation of large sensory-neuron nuclei and permits the analysis of frozen human tissues, although it captures nuclear rather than whole-cell transcriptomes [17]. When combined with spatial transcriptomics and computational approaches, these technologies can identify disease-associated cell states, suggest possible temporal trajectories, and nominate candidate intercellular communication pathways [18].

This review organizes the emerging single-cell evidence around the concept that neuropathic pain involves coordinated cell-state remodeling across the neuronal, glial, immune, and stromal compartments of the DRG. A cell state is defined here as a reproducible transcriptomic configuration within a specified cell population. We first summarize the technological evolution from scRNA-seq to snRNA-seq and spatially informed approaches, and then describe the cellular taxonomy of the DRG across species. We next compare disease-associated remodeling across peripheral nerve injury, diabetic neuropathy, and chemotherapy-induced neuropathy. Finally, we examine the correspondence between experimental findings and emerging human DRG atlases and discuss how these data may support translational interpretation, including target prioritization, mechanistic refinement, biomarker concepts, and analgesic development.

This narrative review was informed by searches of PubMed, Web of Science, and Scopus for studies published between January 2018 and January 2026. Search terms included combinations of “dorsal root ganglion,” “neuropathic pain,” “single-cell RNA sequencing,” “single-nucleus RNA sequencing,” “spatial transcriptomics,” “human DRG,” and “translational.” We prioritized studies that applied sc/snRNA-seq, spatial or multi-omic techniques, human DRG datasets, or functional validation relevant to translational pain research.

## Methodological considerations for single-cell profiling of the DRG

Single-cell profiling of the DRG presents several technical challenges that can affect cell recovery, transcript detection, and biological interpretation. These challenges are particularly important because sensory neurons exhibit substantial size variability and are closely associated with satellite glial cells and other non-neuronal cell populations [19, 20]. In a typical workflow, freshly isolated DRG tissue is dissociated into intact cells for scRNA-seq, whereas nuclei are isolated from fresh or frozen tissue for snRNA-seq. Following barcoding, library preparation, and sequencing, the resulting data undergo quality control, normalization, dimensionality reduction, clustering, cell-type annotation, and differential expression analysis [21].

The choice between scRNA-seq and snRNA-seq involves important trade-offs. scRNA-seq captures whole-cell transcripts but requires enzymatic and mechanical dissociation, which may preferentially exclude large or fragile myelinated neurons and induce stress-response genes such as *Fos* and *Jun* [17, 22]. By contrast, snRNA-seq avoids extensive tissue dissociation, improves the recovery of large neuronal populations, and can be applied to frozen human tissue [23, 24]. However, snRNA-seq captures only nuclear transcripts and may underrepresent cytoplasmic and locally translated mRNAs, particularly those enriched in distal axonal compartments. Recent efforts to address these trade-offs include a single-soma deep-sequencing strategy that combines laser capture microdissection with Smart-seq2 to generate full-length transcriptomic profiles and detect, on average, more than 9,000 unique genes per human DRG neuron, substantially exceeding the sensitivity of standard snRNA-seq approaches [17, 25, 26].

Both scRNA-seq and snRNA-seq largely lose the spatial organization of the DRG. Spatial transcriptomics preserves anatomical context and localizes neuronal–glial units, immune niches, and disease-associated cellular states [27–31]. Integrating spatial transcriptomic and single-cell datasets therefore enhances the interpretation of cell-type-specific changes, although spatial transcriptomic platforms vary in spatial resolution and transcript detection sensitivity.

Cross-study comparisons are also influenced by batch effects arising from differences in sequencing depth, transcript capture efficiency, cell or nucleus recovery, quality-control thresholds, normalization methods, and data-integration strategies. These factors can affect cluster resolution, estimated cell-type proportions, and differential-expression results, particularly when comparing studies that use different pain models, sampling time points, sexes, or species [32–34]. Greater standardization of experimental design, data processing, and cell-type annotation would improve reproducibility and facilitate comparisons across studies.

## Cellular taxonomy of the DRG

**Fig. 1:**
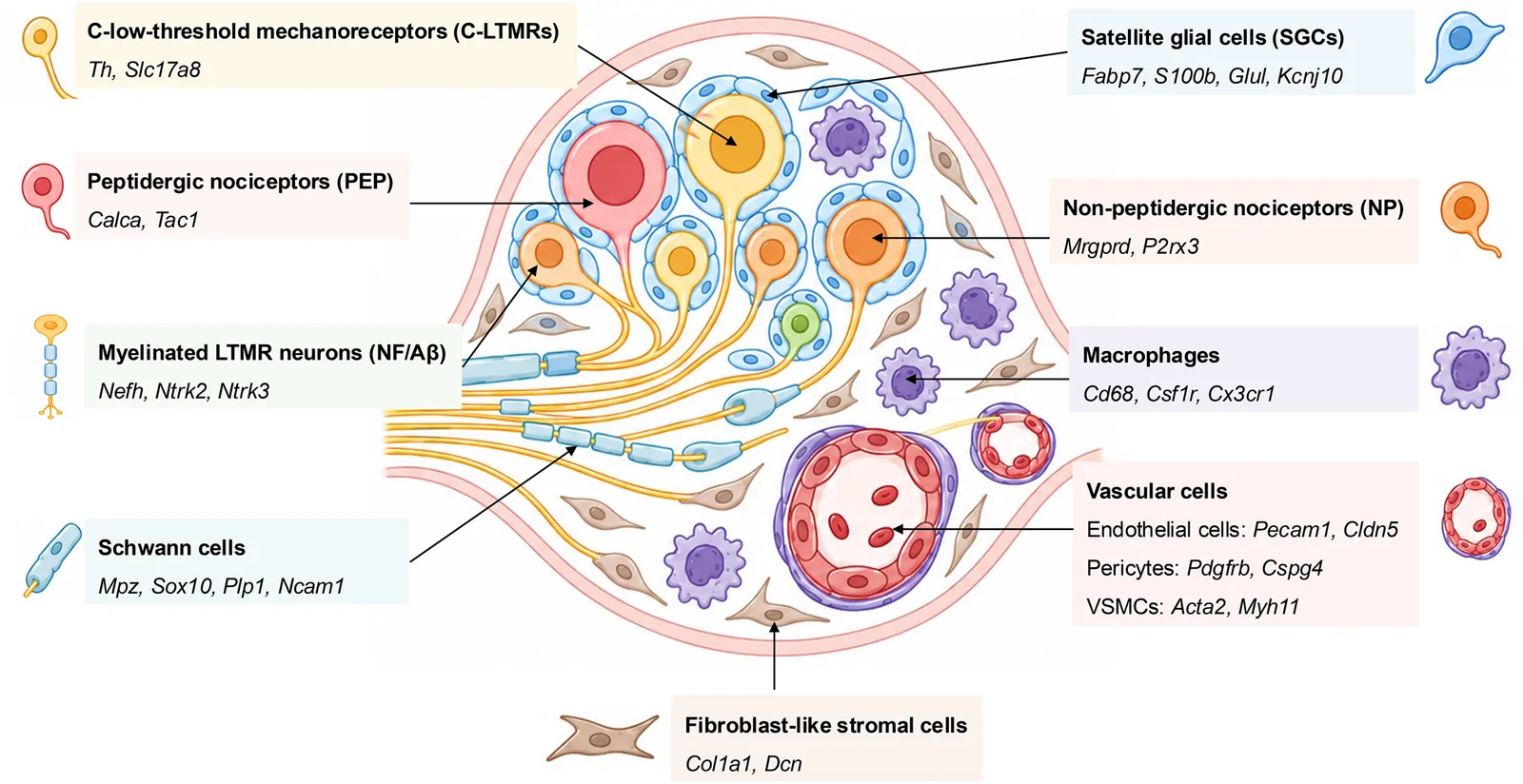
Major cell populations of the dorsal root ganglion and representative marker genes

The DRG comprises a heterogeneous assembly of neuronal and non-neuronal cell populations that function as an integrated unit to maintain somatosensory homeostasis. Recent scRNA-seq and snRNA-seq studies have significantly refined the cellular taxonomy of this structure, revealing greater diversity than was previously recognized using histological or electrophysiological approaches [13, 32, 35]. Fig. 1 presents a schematic overview of this multicellular landscape and its key marker genes, and Table 1 provides a more detailed comparison of marker profiles, functional roles, and human–mouse correspondence.

**Table 1:** Representative DRG cell populations, marker genes, functional roles, and human–mouse correspondence in sc/snRna-seq studies

Cell Type	Population / program	Key Markers (Mouse)	Key Markers (Human)	Main role	Human–mouse correspondence	References
Sensory Neurons	Peptidergic nociceptors (PEP)	*Calca, Tac1, Trpv1, Ntrk1*	*CALCA, TAC1, TRPV1, NTRK1*	Thermal/chemical nociception; CGRP/SP release	Moderate–high; broader TRPV1 distribution in humans	[13, 30, 35, 36]
	Non-peptidergic nociceptors (NP)	*Mrgprd, P2rx3, Ret*	*MRGPRD* (low/diffuse), *P2RX3*	Mechanical nociception and pruriception	Moderate; diffuse MRGPRD expression in humans	[13, 35–37]
	C-low-threshold mechanoreceptors (C-LTMRs)	*Th, Slc17a8 (Vglut3)*	*TH* (low), *SLC17A8/VGLUT3* (variable)	Affective touch; gentle mechanosensation	Moderate; one-to-one physiological correspondence incomplete	[17, 38, 39]
	Myelinated LTMR neurons (NF/Aβ)	*Nefh, Ntrk2, Ntrk3, Ret*	*NEFH, NTRK2, NTRK3*	Low-threshold mechanosensation; touch and vibration	High	[13, 30, 35]
	Proprioceptors	*Pvalb, Ntrk3, Etv1*	*PVALB, NTRK3*	Proprioception and muscle spindle innervation	High	[13, 30, 35]
	Aδ-like myelinated nociceptors / HTMRs	*Nefh, Scn10a, Trpv1*	*NEFH, SCN10A, TRPV1* (partial overlap)	High-threshold mechano-/thermo-nociception	Moderate; correspondence is supported but not one-to-one	[13, 35, 36]
	Human-enriched PIEZO2-high PEP neurons	No clear counterpart	*PIEZO2* (high), *CALCA*	Putative mechanosensory function	Low/absent; no clear murine counterpart	[17]
	Human-enriched *NTRK3/DCC* nociceptor-related neurons	No clear counterpart	*NTRK3, DCC*, opioid-related transcripts	Human-enriched sensory program; function uncertain	Low/absent; persists into adulthood in human DRG	[40]
Satellite Glial Cells	Homeostatic SGCs	*Kcnj10, Fabp7, Glul, Gja1*	*KCNJ10, FABP7, GLUL, GJA1*	Perineuronal ensheathment; K^+^ buffering; glial homeostasis	Moderate; broad identity conserved, fine subtype unresolved	[18, 41, 42]
	Spatially biased SGC programs	*Fabp7, Kcnj10*	Not established	Preferential association with selected neuronal classes	Uncertain; supported mainly by mouse spatial data	[43]
	UGT8+ metabolic SGCs	*Ugt8, GalCer* biosynthesis genes	Not established	GalCer biosynthesis; lipid support at the neuron–glia interface	Uncertain; defined mainly in rodent DPN	[44]
	TIMP3+ SGCs	*Timp3*	*TIMP3*	ECM homeostasis; metalloproteinase restraint	Moderate; human functional validation is limited	[45]
Schwann Cells	Myelinating Schwann cells	*Mpz, Mbp, Prx*	*MPZ, MBP*	Axonal myelination and saltatory conduction	High	[46, 47]
	Non-myelinating Schwann cells	*Ncam1, L1cam, Scn7a*	*NCAM1, L1CAM*	Ensheathment of unmyelinated C-fibers	High	[46, 47]
	Stress response-related Schwann cells (SRSCs)	*Npy, Spp1*	Not established	Injury-associated remodeling	Unknown; described in mouse	[48]
	Repair Schwann cells	*Jun, Ngf, Bdnf*	*JUN, NGF, BDNF*	Injury-induced repair; trophic support	Moderate	[46, 49]
Macrophages	Resident macrophages (sNAMs)	*Cx3cr1, Csf1r, Aif1, Cd68*	*CX3CR1, CSF1R, AIF1*	Tissue surveillance; debris clearance; immune homeostasis	High; broad population conserved	[50–52]
	CCR2+ recruited monocyte/macrophage states	*Ccr2, Trem2, Lgals3*	*CCR2, TREM2, LGALS3*	Context-dependent immune modulation	Moderate/uncertain; mainly mouse peripheral-nerve evidence	[53, 54]
Mast Cells	Mrgprb2+ mast cells	*Kit, Mrgprb2, Tpsb2*	*KIT, MRGPRX2*	Neuropeptide- triggered degranulation; neurogenic inflammation	Moderate; MRGPRX2 is a functional counterpart, not a direct ortholog	[55]
Endothelial Cells	Fenestrated capillary endothelial cells	*Pecam1, Cldn5, Vwf*	*PECAM1, CLDN5, VWF*	Permeable neurovascular unit; nutrient exchange; barrier function	High	[56, 57]
Pericytes	DRG pericytes	*Rgs5, Pdgfrb, Acta2*	*RGS5, PDGFRB, ACTA2*	Capillary support; vascular tone and barrier stability	High	[56–58]
Fibroblast-like stromal cells	Stromal / fibroblast-like cells	*Col1a1, Dcn, Col3a1*	*COL1A1, DCN, COL3A1*	ECM synthesis; structural support and signaling	Moderate-high; broad identity conserved	[59, 60]
	Activated myofibroblast-like cells	*Acta2, Col1a1*	*ACTA2, COL1A1*	Fibrotic ECM deposition after injury	Moderate; direct human DRG evidence limited	[59, 60]

Abbreviations: DRG, dorsal root ganglion; ECM, extracellular matrix; GalCer, galactosylceramide; HTMRs, high-threshold mechanoreceptors; LTMRs, low-threshold mechanoreceptors; sNAMs, sensory neuron-associated macrophages; SGCs, satellite glial cells

### Sensory neurons

Somatosensory neurons in the DRG convert mechanical, thermal, and chemical stimuli into electrical signals transmitted to the spinal dorsal horn. Transcriptomic profiling has resolved these neurons into approximately 14–18 conserved subtypes across species, depending on the sequencing platform and data integration strategy. These subtypes can be broadly grouped into peptidergic nociceptors (PEP), characterized by *Calca* and *Tac1* expressions; non-peptidergic nociceptors (NP), marked by *Mrgprd* and *P2rx3*; and NF/Aβ myelinated low-threshold mechanoreceptor neurons, defined by *Nefh* and *Ntrk2/3* [32, 34, 35, 61, 62]. Additional functional specialization has been identified within these broad categories. C-low-threshold mechanoreceptors (C-LTMRs), defined by *Th* and *Vglut3* co-expression, represent a distinct lineage implicated in affective touch and temperature sensation [38, 39]. At the subcellular level, Landeros et al. described a neuronal membrane proteasome complex on nociceptor surfaces that modulates excitability through extracellular peptide degradation, adding a previously unrecognized layer of regulation [63].

An important consideration for translational research is the incomplete conservation of neuronal subtypes across species. In mice, PEP and NP populations form transcriptionally discrete clusters, whereas human nociceptors exhibit a more continuous transcriptional spectrum [30, 35, 36]. Notably, Yu et al. identified the hPEP.PIEZOh cluster, a human-enriched subtype with high *PIEZO2* expression that lacks a clear murine counterpart and may contribute to visceral mechanosensation [17].

### Satellite glial cells

SGCs form a continuous perineuronal sheath around each sensory neuron soma, creating a restricted microenvironment that supports metabolic coupling, potassium buffering, and neurotransmitter recycling [41, 42, 64]. Although SGCs were traditionally considered as a homogeneous population, recent transcriptomic and spatial analyses have revealed considerable molecular heterogeneity among SGCs. Different SGC subtypes appear to preferentially associate with specific neuronal classes; for example, *Fabp7*-enriched SGCs are predominantly found around large mechanoreceptors, whereas *Kir4.1*-expressing SGCs preferentially ensheathe nociceptors [43]. In addition, SGCs actively participate in lipid metabolism. Xu et al. characterized a subpopulation enriched in enzymes involved in galactosylceramide biosynthesis, including *Ugt8*, indicating that SGCs contribute to the structural integrity of the neuron–glia interface through lipid homeostasis [44]. Under homeostatic conditions, SGCs express high levels of *Timp3*, which helps maintain extracellular matrix stability [45].

### Schwann cells

SCs in the DRG and peripheral nerves are broadly categorized into myelinating (*Mpz+*) and nonmyelinating (*Ncam1+*) subtypes. After nerve injury, SCs can revert to a progenitor-like repair phenotype characterized by the upregulation of Jun and neurotrophic factors, such as *Ngf* and *Bdnf* [46, 47, 49]. Recent single-cell studies have further expanded this classification. Lan et al. identified a subtype termed stress response–related Schwann cells, characterized by high neuropeptide Y (*Npy*) expression, that functions as a mechanical sensor coordinating tissue remodeling through osteopontin (*Spp1*) signaling [48]. Furthermore, Li et al. provided evidence that specific SC subpopulations regulate the local inflammatory environment through interactions with macrophages, indicating a more active role in immune regulation than was previously recognized [65].

### Immune cells

The DRG maintains a resident immune niche composed primarily of macrophages, often referred to as sensory neuron–associated macrophages. Single-cell profiling has shown that these macrophages exist along a spectrum of activation states rather than conforming to the classical M1/M2 dichotomy [50–52]. Their functions may depending on the type of injury, disease stage, and local microenvironment. Other immune cell populations may also contribute to DRG remodeling. For example, mast-cell activation through *Mrgprb2* promotes neurogenic inflammation and pain in selected nerve-injury models [55]. Collectively, these findings underscore the functional heterogeneity of immune responses associated with neuropathic pain.

### Vascular and stromal cells

The vascular compartment of the DRG consists of fenestrated endothelial cells (*Pecam1+*) and pericytes (*Rgs5+*, *Pdgfrb+*), which collectively form a neurovascular unit that is more permeable than the blood–brain barrier [56]. Under metabolic stress, such as hyperglycemia, reduced mural cell support and disruption of endothelial tight junctions may impair barrier function and facilitate immune cell infiltration [57, 58]. Fibroblasts within the DRG and peripheral nerve synthesize extracellular matrix components, and transcriptomic studies have distinguished epineurial, endoneurial, and related stromal subpopulations, some of which can adopt a pro-fibrotic myofibroblast-like state characterized by *Acta2* expression under chronic pain or injury conditions [59, 60].

## Etiology-specific DRG cell-state reprogramming

**Fig. 2:**
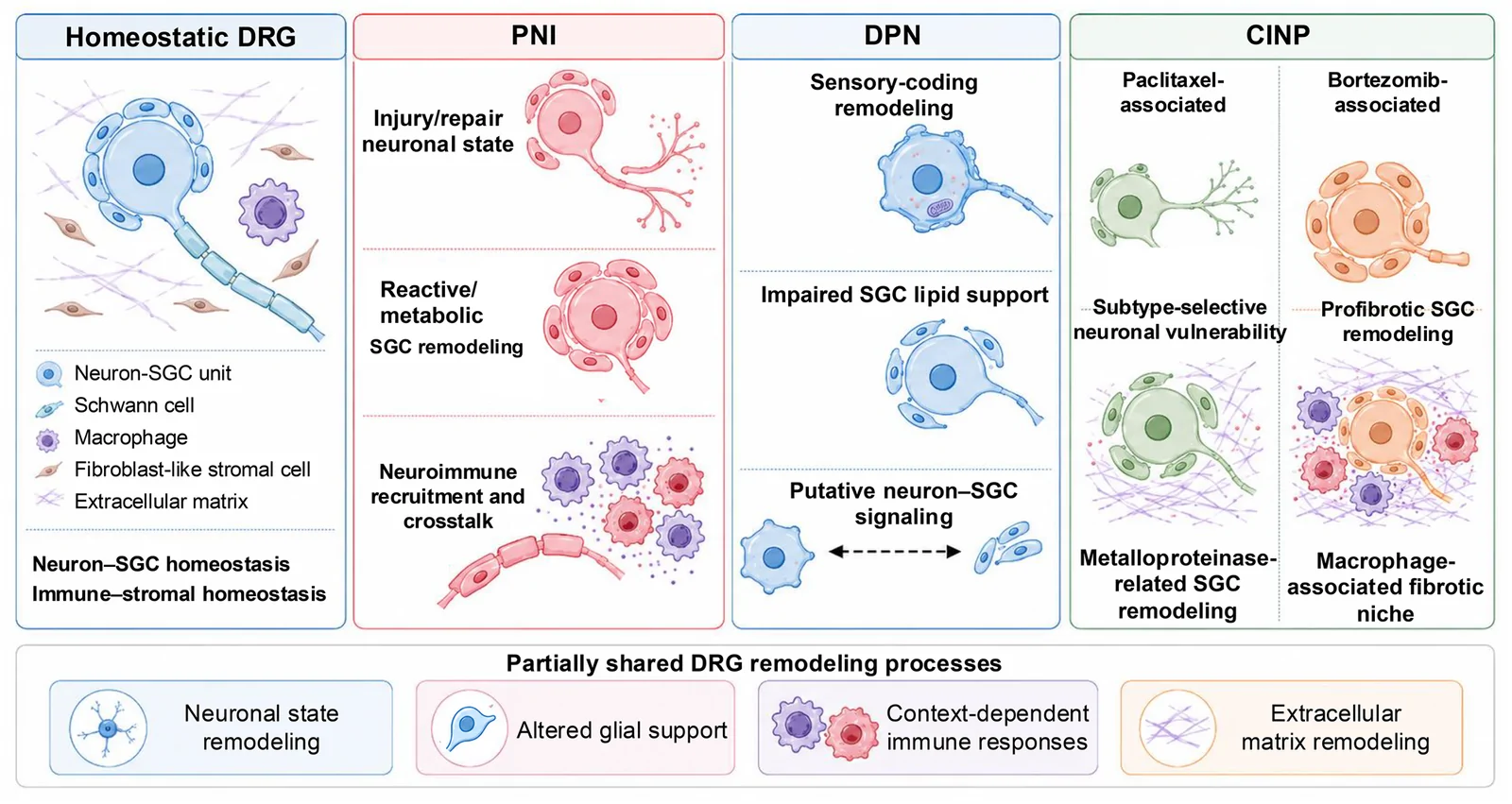
Shared and etiology-associated DRG cell-state remodeling in neuropathic pain

Building on the cellular taxonomy described above, this section examines how distinct neuropathic pain etiologies reshape cellular states within the DRG. Single-cell and single-nucleus studies show that peripheral nerve injury, diabetic neuropathy, and chemotherapy-induced neuropathy share several injury-response programs, but also display distinct neuronal, glial, immune, and stromal signatures. Table 2 summarizes shared and etiology-associated DRG cell-state programs and their supporting evidence, and Figure 2 provides a schematic comparison of homeostatic and disease-associated remodeling across these etiologies.

**Table 2:** Shared and etiology-associated DRG cell-state programs in neuropathic pain

Etiology/model	Main compartment	Pattern	Cell-state program or process	Key features	Evidence	References
PNI	Injured sensory neurons	Shared; PNI-associated	Injury/repair neuronal state	*Atf3*, *Sprr1a*, *Gap43* ↑; subtype markers such as *Tac1*, *Mrgprd*, or *Nefh* ↓	Transcriptomic; selected functional support	[66, 67]
PNI	Satellite glial cells	Shared glial remodeling; PNI-associated	*Reactive/metabolic SGC remodeling*	Gfap, Apoe ↑; Kcnj10, Hmgcs1, Fdft1 ↓	Transcriptomic; selected functional support	[68–70]
PNI	Injured neurons, Schwann cells, macrophages	Shared neuroimmune process; PNI-associated	*Neuroimmune recruitment and crosstalk*	*Jun/Npy-*associated Schwann-cell states*; Il34–Csf1r, Ccl2–Ccr2, and Csf1–Csf1r*signaling	*Transcriptomic + functional (animal)*	[48, 49, 53, 65, 71]
PNI	Mast cells	PNI-associated	Neuropeptide-responsive mast-cell activation	*Mrgprb2*	Functional (animal; selected models)	[55]
DPN	Sensory neurons	Shared neuronal remodeling; DPN-associated	Sensory-coding remodeling	*Fxyd7*, *Atp1b1*, and *Mrgprd* ↑in selected neurons	Transcriptomic + functional (animal)	[37, 72]
DPN	Satellite glial cells	Shared loss of glial support; DPN-associated	*Impaired SGC lipid support*	*Ugt8* expression and *GalCer biosynthesis* ↓	Transcriptomic + functional (animal)	[44]
DPN	Satellite glial cells and sensory neurons	DPN-associated	*Putative neuron–SGC signaling*	*Ptn–Ncl* and *Mdk–Ncl*	Computational inference	[73]
Human diabetic peripheral neuropathy	Satellite glial cells and non-myelinating Schwanncells	Human disease-associated	*Glial remodeling with neurodegeneration*	*SPP1*+ Nageotte nodules associated with dystrophic or sprouting axons	Human spatial + histopathological	[31]
Paclitaxel-induced CINP	Sensory neurons, including C-LTMR-like neurons	Paclitaxel-associated	Subtype-selective neuronal vulnerability	Camk1d ↑ across several neuronal subtypes; prominent remodeling of Th/Slc17a8-defined neurons	Transcriptomic + functional (animal)	[74]
Paclitaxel-induced CINP	Satellite glial cells	Paclitaxel-associated	Metalloproteinase-related SGC remodeling	*Timp3* ↓; altered *Mmp14* and *Adam17* signaling	Transcriptomic + functional (animal)	[45]
Bortezomib-induced CINP	Satellite glial cells	*Bortezomib- and sex-associated*	Profibrotic SGC remodeling	*Atf3*-associated fibrotic and ECM programs	Transcriptomic (animal; sex-associated)	[75]
Bortezomib-induced CINP	Satellite glial cells, macrophages, stromal cells	Bortezomib-associated fibrotic remodeling	Macrophage-stromal fibrotic niche	Macrophage expansion, collagen deposition, and ECM remodeling	Transcriptomic + depletion evidence (animal)	[75]

**Abbreviations:** CINP, chemotherapy-induced neuropathic pain; DPN, diabetic painful neuropathy; DRG, dorsal root ganglion; ECM, extracellular matrix; GalCer, galactosylceramide; PNI, peripheral nerve injury; SGC, satellite glial cell

### Peripheral nerve injury

Peripheral nerve injury (PNI) is a leading cause of neuropathic pain, frequently resulting from trauma, compression, or surgical complications [76–78]. Preclinical models, including chronic constriction injury, spared nerve injury, and spinal nerve ligation, have been instrumental in elucidating the mechanisms underlying the transition from acute trauma to chronic pain [79, 80]. Recent sc/snRNA-seq studies indicate that PNI induces coordinated transcriptional remodeling across neurons, satellite glial cells, Schwann cells, and immune cell populations within the DRG [81].

At the neuronal level, PNI is characterized by a partial loss of subtype identity and activation of injury-response programs. Injured sensory neurons exhibit reduced expression of several subtype-defining genes accompanied by robust induction of *Atf3*, although the specific transcriptional changes vary across neuronal subtypes, injury models, and sampling time points [66]. *Atf3*-positive neurons also express regeneration-associated genes, including *Sprr1a* and *Gap43*, suggesting activation of an axonal repair program [67]. Other injury-induced signals may link repair and pain modulation. *Adcyap1*, which encodes PACAP, has been proposed to promote axon regeneration while limiting nociceptive signaling after PNI [82]. *Clcf1* has also been identified in injury-induced neuronal clusters and may connect peripheral neuronal reprogramming with spinal astrocyte activation and central sensitization [16]. In addition, the iPain atlas identified a senescence-like nociceptor state in selected chronic pain models, and senolytic treatment reduced pain-related behaviors in mice [81]. However, behavioral improvement alone does not demonstrate that the underlying transcriptional state itself is reversible or remains stable across disease stages. These findings suggest that neuronal injury states comprise both adaptive repair responses and maladaptive programs that contribute to persistent pain.

Non-neuronal cells also undergo extensive remodeling following PNI. Satellite glial cells exhibit increased expression of reactive genes, including *Gfap* and *Apoe*, accompanied by reduced expression of homeostatic genes such as *Kcnj10* [68, 69]. Downregulation of cholesterol biosynthesis genes, including *Hmgcs1* and *Fdft1*, may further impair lipid support for neuronal repair [69, 70]. Similarly, Schwann cells display injury-induced plasticity, including repair-associated and stress-related states [83]. Recent evidence suggests that *Il34/Csf1r*-mediated Schwann cell–macrophage communication may sustain neuroinflammation and chronic pain after nerve injury [65].

The immune response in PNI follows a sequential pattern. In the acute phase, injured neurons secrete chemokines, including *Ccl2* and *Csf1*, promoting the infiltration of *Ccr2*+ monocyte–derived macrophages [53, 71]. These macrophages may sustain a pro-inflammatory DRG milieu, although their functions are likely to be context- and time-dependent. Mast-cell activation through *Mrgprb2* has also been implicated in neurogenic inflammation and pain after nerve injury [55]. Together, these findings suggest that PNI is not only a disorder of damaged axons, but also a failure of multicellular homeostasis within the DRG.

### Diabetic painful neuropathy

Diabetic painful neuropathy (DPN) is the most common chronic complication of diabetes, affecting up to half of all people with diabetes, of whom a substantial subset develops neuropathic pain. [84, 85]. Unlike the acute mechanical insult of PNI, DPN develops under chronic metabolic stress caused by hyperglycemia, dyslipidemia, oxidative stress, and microvascular dysfunction [86]. Single-cell studies suggest that this metabolic context produces a pattern of DRG remodeling that differs from that induced by traumatic nerve injury.

At the neuronal level, single-cell profiling has identified disease-specific subpopulations associated with DPN. Zhou et al. described mechanical allodynia-associated clusters defined by *Fxyd7* and *Atp1b1* upregulation, implicating ionic dysregulation in the maintenance of mechanical hypersensitivity [72]. George et al. reported aberrant *Mrgprd* upregulation in nonpeptidergic neurons in diabetic neuropathy, suggesting a role for ectopic activation of cutaneous sensory afferents in painful phenotypes [37]. Altered expression of mechanosensitive and voltage-gated ion channels, including *Piezo2*, *Scn9a*, and *Scn3a*, may further contribute to sensory abnormalities in DPN [86]. These findings suggest that DPN-related neuronal remodeling is driven primarily by metabolic stress rather than by a classic axotomy-like injury program alone.

A distinguishing feature of DPN at the single-cell level is the metabolic impairment of SGCs. Reduced *Ugt8* expression and impaired galactosylceramide biosynthesis disrupt lipid raft integrity and weaken SGC–neuron metabolic coupling, thereby contributing to nociceptor hyperactivity [44]. Inferred ligand–receptor analyses have also suggested altered *Ptn–Ncl* and *Mdk–Ncl* signaling between glial cells and neurons [72, 73]. This metabolic uncoupling contrasts with the more inflammatory gliosis typically observed after PNI. Human DPN tissue provides additional evidence of advanced DRG remodeling. Spatial analysis of Nageotte nodules showed that these structures are mainly composed of SPP1-expressing satellite glial cells and non-myelinating Schwann cells associated with dystrophic or sprouting sensory axons [31]. This finding provides a human pathological correlate of multicellular DRG degeneration and likely reflects relatively advanced neurodegenerative remodeling in DPN.

The immune response in DPN also appears to be context-dependent. Ccr2-positive macrophages in prediabetic mice can express *Trem2* and *Lgals3* and may delay terminal sensory axon degeneration through galectin 3 signaling [54]. This contrasts with the predominantly pain-promoting macrophage response described in some PNI models and suggests that indiscriminate anti-inflammatory strategies may not be appropriate across all neuropathic pain etiologies [53, 54].

### Chemotherapy-induced neuropathic pain

Chemotherapy-induced neuropathic pain (CINP) is a common, dose-limiting adverse effect of several antineoplastic agents [87]. Unlike PNI and DPN, CINP results from drug-induced neurotoxicity, including mitochondrial dysfunction, impaired axonal transport, and altered neuroimmune signaling [88]. Single-cell studies further suggest that CINP is not a uniform syndrome but instead comprises agent-specific patterns of neuronal and non-neuronal vulnerability.

In paclitaxel-induced neuropathy, Sun et al. identified widespread *Camk1d* upregulation across several sensory-neuron subtypes, together with prominent transcriptomic remodeling of *Th/Slc17a8*-defined C-LTMR-like neurons. Functional knockdown experiments implicated *Camk1d* in thermal and cold hypersensitivity, but did not demonstrate that its expression or function was restricted to the C-LTMR-like population [74]. Concurrently, Tonello et al. showed that paclitaxel reduces *Timp3* expression in satellite glial cells, leading to disinhibition of metalloproteinase-related signaling involving *Mmp14* and *Adam17* and contributing to a pro-inflammatory microenvironment [45]. These findings suggest that paclitaxel-induced neuropathy involves both neuronal subtype vulnerability and glial microenvironmental remodeling.

Bortezomib-induced neuropathy appears to involve a distinct glial and stromal response. Thomsen et al. reported that male mice develop a specific *Atf3*-driven profibrotic program in SGCs after bortezomib treatment, resulting in pathological collagen deposition that physically constricts sensory neurons [75]. This response was linked in part to macrophage–SGC crosstalk and was less prominent in female mice, which showed relative enrichment of lipid metabolic programs [75]. These findings highlight sex as an important biological variable in CINP and suggest that different chemotherapeutic agents may engage distinct DRG pathological programs within the DRG.

Single-cell studies reveal distinct, treatment-associated DRG responses to different chemotherapeutic agents. Paclitaxel-induced neuropathy is associated with C-LTMR-like neuronal vulnerability and SGC metalloproteinase signaling, whereas bortezomib-induced neuropathy appears to involve sex-dependent neurofibrotic remodeling [45, 74, 75]. These differences support the view that CINP is not a uniform neurotoxic syndrome, but rather a group of agent-specific pathobiological states that may require mechanism-tailored therapeutic strategies.

### Shared and etiology-specific DRG remodeling

Comparisons across PNI, DPN, and CINP indicate partially shared biological processes rather than a single conserved molecular program. A cell state refers here to a reproducible transcriptomic configuration within a defined cell population, whereas a cell-state transition requires evidence of temporal progression. Because most current studies rely on single-time-point or repeated cross-sectional sampling, they identify disease-associated cell states and potential temporal patterns rather than directly tracking transitions within the same cells [44, 45, 66, 72, 75].

The principal overlap across etiologies occurs at the level of broad biological processes, including neuronal state remodeling, altered glial homeostatic support, context-dependent immune responses, and extracellular matrix remodeling [45, 53, 89]. These cell states may serve adaptive, maladaptive, or degenerative functions depending on the cell type, disease stage, and experimental context. For example, injury-associated neuronal programs may comprise both regenerative and pain- associated components [66], whereas Nageotte nodules in human diabetic peripheral neuropathy are more consistent with advanced neurodegenerative remodeling than with an early or pain-specific mechanism [31]. Accordingly, similar transcriptomic features across etiologies should not be assumed to produce equivalent functional outcomes.

Etiology-specific patterns remain prominent. PNI is characterized by neuronal injury and repair states, reactive and metabolic SGC remodeling, and neuroimmune cell recruitment. DPN is associated with altered sensory coding, impaired lipid-support functions in SGCs, and inferred neuron–SGC signaling [66, 90, 91]. CINP exhibits substantial agent-associated variation, with subtype-selective neuronal vulnerability and metalloproteinase-dysregulated SGC states following paclitaxel treatment, as well as male-biased profibrotic remodeling following bortezomib treatment. However, the temporal coordination of these responses remains incompletely understood. Available evidence suggests that PNI begins with early neuronal injury responses and chemokine induction, followed by glial and immune remodeling, whereas metabolic, neuronal, and glial abnormalities in DPN may evolve in parallel [44, 72]. In CINP, the timing and persistence of these responses vary according to the chemotherapeutic agent and biological context. Collectively, current evidence supports etiology-specific temporal models rather than a unified sequence of DRG remodeling [45, 74].

Large-diameter myelinated neurons, including NF mechanoreceptors and proprioceptors, remain less well characterized than nociceptors in disease-focused datasets. Altered *Nefh*-expressing populations have been reported following PNI; however, their contribution to persistent pain remains uncertain [66]. Comparable analyses in DPN and CINP remain limited. The C-LTMR-like population implicated in paclitaxel-induced neuropathy represents a distinct unmyelinated mechanoreceptive lineage and should not be interpreted as evidence for the involvement of large myelinated neurons [38, 74]. Thus, the improved recovery of large neuronal nuclei by snRNA-seq has not yet translated into a clear understanding of their disease-specific roles.

## Single-cell profiling of the human DRG

The limited translational success of analgesic candidates identified in rodent models has underscored the need to define the molecular architecture of the human DRG at cellular resolution [92]. Recent single-nucleus RNA sequencing, spatial transcriptomics, single-soma deep RNA sequencing, and cross-species integration studies have generated increasingly comprehensive human DRG atlases [30, 35]. These datasets demonstrate broad conservation of the major sensory neuron classes while also revealing species-specific differences in neuronal subtype organization, glial programs, and the distribution of candidate therapeutic targets. Table 3 summarizes key human DRG evidence, relevant rodent comparisons, translational implications, and major caveats.

**Table 3:** Human DRG evidence and translational implications

Human evidence	Representative findings	Rodent comparison / context	Translational implication	Key caveat	References
Nociceptor architecture and marker distribution	Human nociceptors show a more continuous transcriptional spectrum, broader *TRPV1* distribution and lower or more diffuse MRGPRD expression	Mouse peptidergic and nonpeptidergic nociceptors form more discrete clusters	Rodent subtype maps and target distributions require cautious human extrapolation	Donor/postmortem atlases do not necessarily reflect protein function, excitability, or treatment response	[30, 34–36]
Human-enriched neuronal populations	PIEZO2-high peptidergic neurons and *NTRK3/DCC*-expressing nociceptor-related neurons have been identified in human DRG	No clear one-to-one adult mouse counterpart	Human-enriched sensory programs for target discovery	Functional roles and pain-state relevance remain unresolved	[17, 40]
Human glial programs	Human SGCs and Schwann cells show species-divergent neurotrophic, ECM, and inflammatory expression programs, including NGF-related patterns	Direct correspondence with rodent glial programs remains uncertain	Human cellular context for glial mechanisms and candidate targets	Expression patterns alone do not establish clinical causality or therapeutic relevance	[18]
Human disease pathology	Nageotte nodules in diabetic peripheral neuropathy contain *SPP1*-expressing SGCs and non-myelinating Schwann cells associated with dystrophic or sprouting axons	Not fully captured by standard rodent diabetic neuropathy models	Direct human tissue evidence of multicellular DRG remodeling and neurodegeneration	May represent advanced pathology rather than an early, reversible, or pain-specific state	[31]
Integration with genetic evidence	GWAS-supported pain candidates, such as *SCN11A* and *EFNB2* can be mapped to human DRG cell populations	Genetic signals can be integrated with cell-type-resolved human DRG expression	Prioritization of genetically anchored human pain candidates	Genetic and cellular anchoring does not establish functional validation or therapeutic tractability	[93]

Abbreviations: DRG, dorsal root ganglion; ECM, extracellular matrix; GWAS, genome-wide association study; SGCs, satellite glial cells

### Human DRG atlases

Human DRG profiling has evolved from neuronal taxonomy toward integrated and spatially informed reference atlases. Nguyen et al. used snRNA-seq to classify human DRG neurons into major sensory subtypes, providing an initial framework for comparisons with rodent sensory neurons [35]. Tavares-Ferreira et al. complemented this approach by combining snRNA-seq with spatial transcriptomics, thereby preserving anatomical context and identifying molecular signatures of human nociceptors within intact DRG sections [30]. Yu et al. applied single-soma deep RNA sequencing to individual human DRG neurons, improving transcript detection and revealing neuronal diversity that may be underrepresented by nuclear profiling alone [17]. Cross-species integration efforts, such as the harmonized trigeminal and DRG atlas generated by Bhuiyan et al., further provide standardized annotations for comparing neuronal and non-neuronal cell populations across species [34].

The primary value of these atlases is that they provide a human molecular framework for interpreting findings from rodent models. They help determine whether candidate molecular targets or cell-state programs are present in the human DRG, whether they are enriched in nociceptors or non-neuronal cell populations, and whether their cellular distribution is conserved across species.

### Cross-species differences in DRG cell populations

Species differences are evident not only in neuronal populations but also in glial and immune cell populations. At the neuronal level, human nociceptor populations appear more transcriptionally continuous than their murine counterparts. Markers such as *TRPV1* and *MRGPRD*, which help distinguish peptidergic and non-peptidergic nociceptor populations in rodents, exhibit broader and less discrete expression patterns in the human DRG [30, 32]. Some human-enriched neuronal populations, including *PIEZO2*-high peptidergic neurons and *NTRK3/DCC*-expressing nociceptor-related neurons [35, 40], also lack clear one-to-one correspondence with mouse subtypes. These differences have important implications for analgesic development, because molecular targets that appear restricted to discrete nociceptor populations in mice may be more broadly expressed or differently distributed in humans.

Cross-species differences also extend to satellite glial cells and Schwann cells [94]. Human glial populations exhibit distinct neurotrophic, extracellular matrix, and inflammatory gene expression profiles, including prominent NGF-related signaling in satellite glial cells [18]. These findings help define the human cellular context of candidate molecular targets but do not establish their roles in clinical pain. Consequently, glial and immune mechanisms identified in rodent models require validation in human DRG tissue.

### Human DRG evidence in neuropathic pain

Disease- focused human DRG studies remain limited. Spatial profiling of Nageotte nodules in diabetic peripheral neuropathy identified *SPP1*-expressing satellite glial cells and non-myelinating Schwann cells associated with neuronal loss and dystrophic or sprouting sensory axons [31]. These findings provide direct evidence of multicellular DRG remodeling in human disease, but likely reflect advanced neurodegenerative pathology rather than an early or pain-specific mechanism. Bulk transcriptomic analyses of clinically phenotyped human DRG samples have also identified sex-associated neuroimmune signatures [33]. However, the cellular origins of these signatures require confirmation using single-cell or spatial transcriptomic approaches.

Most human DRG single-cell datasets are derived from organ donors or postmortem specimens with incomplete pain phenotyping. Donor age, sex, comorbidities, medication exposure, postmortem interval, tissue preservation, and agonal state may all influence transcriptomic profiles [95, 96]. Moreover, transcript abundance does not necessarily predict protein expression, neuronal excitability, or clinical pain phenotypes [97]. The cross-sectional design of current datasets also limits the ability to distinguish among causal, compensatory, and degenerative cell states.

## Translational prospects

**Fig. 3:**
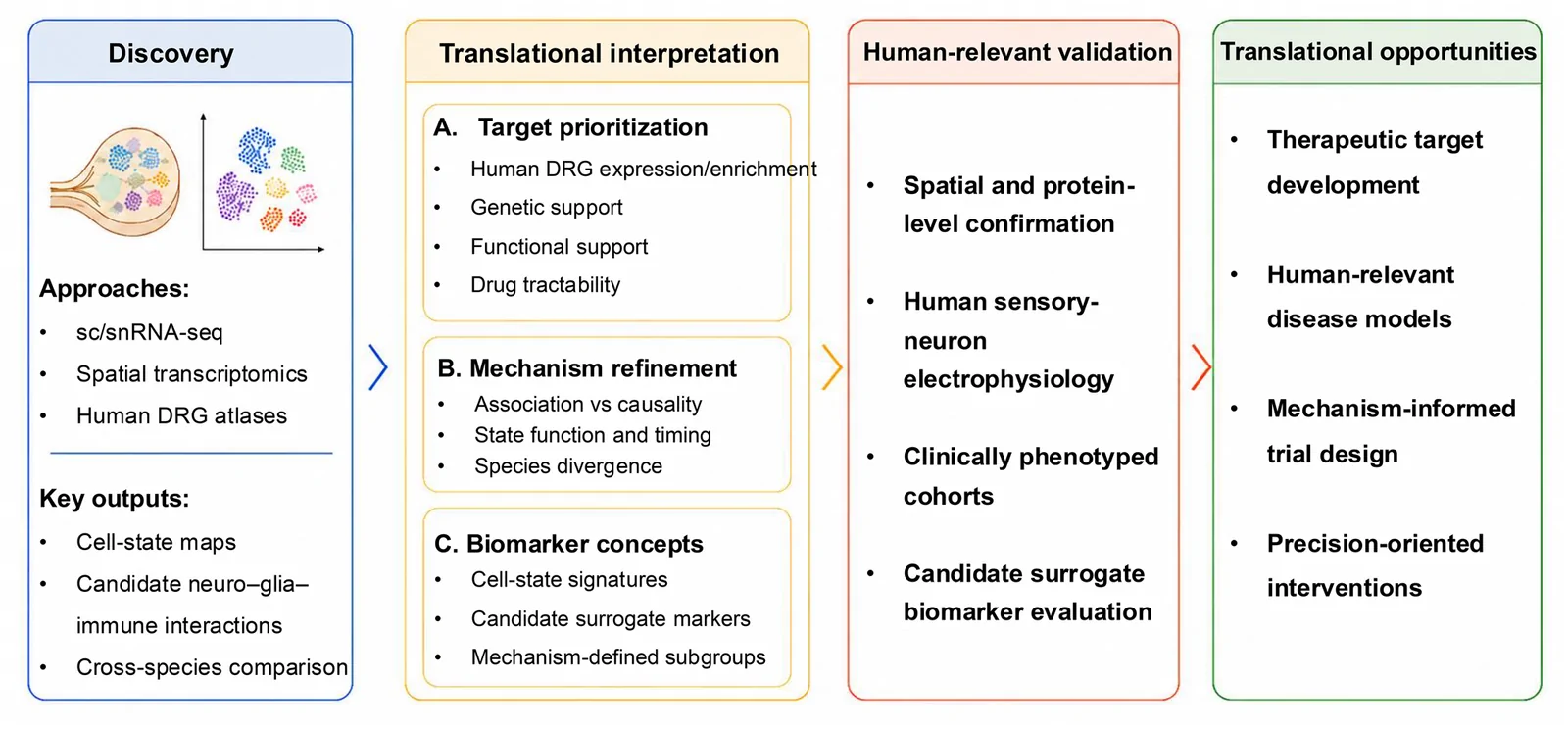
Translational framework for DRG single-cell discoveries in neuropathic pain

Translation of DRG single-cell discoveries requires a stepwise framework that moves from transcriptomic association to translational interpretation, human-relevant validation, and clinically tractable opportunities. Figure 3 summarizes this translational framework.

### Target prioritization

Therapeutic candidates identified through DRG single-cell studies vary considerably in the strength of supporting evidence. Target prioritization should integrate cell-type-resolved human DRG expression with human genetic evidence, functional studies in human sensory neurons, experimental perturbation, and clinical pharmacology. Integration of GWAS data can link candidate genes and cell populations to human pain susceptibility [98, 99], whereas studies using native or cultured human sensory neurons can determine whether transcriptomically nominated targets alter neuronal excitability or other pain-relevant phenotypes. Candidates supported only by differential expression or computational inference should be regarded as hypothesis-generating.

Nav1.8, encoded by *SCN10A*, illustrates a relatively advanced example of target prioritization. Nav1.8 is enriched in nociceptor populations and is preferentially expressed in peripheral sensory neurons, providing a biological rationale for the development of peripherally acting analgesics [100]. This rationale is supported by randomized clinical trials of the selective Nav1.8 inhibitor suzetrigine in acute postoperative pain [101], leading to regulatory approval for the treatment of moderate-to-severe acute pain in adults. However, these findings should not be extrapolated to neuropathic pain, for which the clinical efficacy of Nav1.8 inhibition remains under investigation. Accordingly, Nav1.8 should be regarded as a clinically validated target for acute pain and as a promising, but not yet established, target for peripheral neuropathic pain.

Nav1.7, encoded by *SCN9A*, represents a more complex translational example. Human loss-of-function variants in *SCN9A* cause congenital insensitivity to pain [102], providing strong genetic evidence supporting Nav1.7 as a pain-related target. Nevertheless, some selective Nav1.7 inhibitors have demonstrated limited clinical efficacy [103]. Human DRG transcriptomic studies indicate that *SCN9A* expression is enriched in selected nociceptor populations rather than being uniformly distributed across all sensory neurons [30]. This observation raises the possibility that molecular heterogeneity across patients and pain conditions may have diluted treatment effects in previous clinical trials. However, this interpretation remains hypothetical and requires prospective validation in clinically defined cohorts using target-relevant biomarkers.

Integrating GWAS data with human DRG single-cell datasets provides an additional strategy for to target identification. *SCN11A* and *EFNB2* are enriched in pain-associated human DRG neuronal populations and can therefore be considered genetically anchored candidate genes [93]. However, their causal roles and therapeutic tractability remain uncertain. ERO1 has stronger functional support. Its inhibition reduces excitability in cultured human DRG neurons and attenuates acute pain-like behavior in mice [104]. Whether this mechanism contributes to chronic neuropathic pain remains unresolved.

Several additional pathways remain at the preclinical stage, including Camk1d-associated neuronal vulnerability in paclitaxel-induced neuropathy [74], *Ugt8/GalCer*-related SGC–neuron metabolic uncoupling in diabetic neuropathy [44], *Timp3*-associated extracellular matrix remodeling [45], and *Il34/Csf1r*-mediated Schwann cell–macrophage communication following nerve injury [11]. These findings provide mechanistic insights, however, their translational potential will depend on conservation in the human DRG, a demonstrated causal contribution to pain maintenance, and therapeutic tractability in defined patient subgroups.

### Translational challenges and validation

Candidate cell-state signatures may eventually support mechanism-based patient stratification; however, their current clinical utility remains limited. *ATF3/Atf3*-associated injury-state neurons, senescence-like nociceptor states, and *CCR2/Ccr2*- and *LGALS3/Lgals3*-associated macrophage responses may define biologically informative cell states in experimental models [53, 66, 105]. However, the human DRG cannot be routinely sampled in living patients, and no validated surrogate assay currently captures DRG cell states with sufficient reliability for clinical stratification. Potential surrogates, including skin-biopsy-derived molecular profiles, circulating extracellular vesicles, serum proteins, imaging correlates, and organoid-informed response predictors, should therefore be regarded as future research directions rather than as near-term clinical biomarkers [106, 107].

Emerging experimental platforms may help address part of this validation gap; however, they also require cautious interpretation. Human sensory organoids provide a useful system for testing human-relevant sensory neuron programs and candidate targets [108]. However, most current organoid models resemble developmental sensory states and do not fully recapitulate the architecture of the mature adult DRG, including neuron–satellite glial organization, the vascular niche, immune components, and the pathological features of chronic neuropathic pain. Similarly, nociceptor-selective AAV strategies and promoter-guided gene delivery offer promising approaches for cell-type-selective intervention; however, delivery specificity, tissue tropism, durability of expression, immunogenicity, off-target effects, and long-term safety remain unresolved [109].

## Limitations and interpretive challenges

Technical variation in tissue processing, transcript capture, sequencing depth, and data integration can affect cell recovery, cluster definition, and cross-study comparability. These effects are particularly relevant when comparing datasets generated using different platforms, species, pain models, and sampling time points.

A more fundamental limitation is that current single-cell studies cannot directly establish temporal progression or causal mechanisms. Clustering and differential-expression analyses identify disease-associated transcriptional states but do not establish causality [110]. Pseudotime and trajectory inference methods order cells according to transcriptional states and can generate hypotheses regarding state progression. However, pseudotime is not equivalent to biological time, and most DRG datasets do not track the same cells longitudinally [111]. Even studies that include multiple sampling time points generally provide repeated cross-sectional snapshots. Accordingly, demonstrated cell-state transitions require support from time-resolved sampling, lineage tracing, live-cell imaging, perturbation experiments, or other forms of direct temporal evidence. Likewise, ligand–receptor inference methods predict potential cell–cell communication based on transcript co-expression and curated interaction databases; however, these predictions do not establish spatial proximity, receptor activation, or functional signaling [112]. Furthermore, transcript abundance also does not necessarily predict protein expression, ion-channel activity, neuronal excitability, or pain-related behavior.

Human DRG studies provide important translational context but remain constrained by limited tissue availability, donor heterogeneity, postmortem effects, and incomplete clinical pain phenotyping [30, 35]. Consequently, spatial localization, protein-level characterization, functional perturbation, electrophysiological validation, and clinically annotated human datasets are required before transcriptomic findings can be considered causal or therapeutically relevant.

## Conclusions and future directions

Single-cell and single-nucleus transcriptomics have revealed that neuropathic pain is associated with coordinated remodeling across neuronal, glial, immune, and stromal cell populations within the DRG. Although PNI, DPN, and CINP share broad biological processes, including neuronal state remodeling, altered glial homeostatic support, context-dependent immune responses, and extracellular matrix remodeling, these processes are mediated through distinct cell types, molecular programs, and disease contexts.

Human DRG studies provide an essential framework for evaluating the conservation and cellular context of findings from experimental models. Future studies should integrate longitudinal and spatial transcriptomic profiling with multi-omic, protein-level, and functional validation in clinically annotated human tissues and more mature human-relevant models. Together, these complementary approaches may help distinguish disease-associated transcriptional states from causal mechanisms and identify therapeutically tractable targets for mechanism-based, cell-type-informed analgesic development.

## Data Availability

Not applicable.

## Abbreviations

DRG: Dorsal root ganglion
Atf3: Activating transcription factor 3
SGCs: Satellite glial cells
scRNA-seq: Single-cell RNA sequencing
snRNA-seq: Single-nucleus RNA sequencing
sc/snRNA-seq: Single-cell and single-nucleus RNA sequencing
mRNA: Messenger RNA
ST: Spatial transcriptomics
PEP: Peptidergic nociceptors
Calca: Calcitonin related polypeptide alpha
Tac1: Tachykinin 1
NP: Nonpeptidergic nociceptors
Mrgprd/MRGPRD: MAS-related G protein-coupled receptor D
P2rx3: Purinergic receptor P2rx3
NF: Myelinated low-threshold mechanoreceptors
Nefh: Neurofilament heavy chain
Ntrk2/3: Neurotrophic receptor tyrosine kinase 2/3
C-LTMRs: C-low-threshold mechanoreceptors
Th: Tyrosine hydroxylase
Vglut3: Vesicular glutamate transporter 3
hPEP.PIEZOh: Human PIEZO2-high peptidergic nociceptor subtype
PIEZO2/Piezo2: Piezo type mechanosensitive ion channel component 2
Fabp7: Fatty acid binding protein 7
Kir4.1: Inwardly rectifying potassium channel 4.1
GalCer: Galactosylceramide
Ugt8: UDP glycosyltransferase 8
Timp3: TIMP metallopeptidase inhibitor 3
SCs: Schwann cells
Mpz: Myelin protein zero
Ncam1: Neural cell adhesion molecule 1
Ngf/NGF: Nerve growth factor
Bdnf: Brain-derived neurotrophic factor
Npy: Neuropeptide Y
Spp1/SPP1: Secreted phosphoprotein 1
CCR2/Ccr2: C-C motif chemokine receptor 2
Trem2: Triggering receptor expressed on myeloid cells 2
Lgals3: Galectin 3
Mrgprb2: MAS-related G protein-coupled receptor member b2
Pecam1: Platelet and endothelial cell adhesion molecule 1
Rgs5: Regulator of G protein signaling 5
Pdgfrb: Platelet-derived growth factor receptor beta
Cldn5: Claudin 5
Prg4: Proteoglycan 4
Acta2: Actin alpha 2, smooth muscle
PNI: Peripheral nerve injury
Sprr1a: Small proline-rich protein 1A
Gap43: Growth associated protein 43
Adcyap1: Adenylate cyclase activating polypeptide 1
PACAP: Pituitary adenylate cyclase-activating polypeptide
PAC1R: Pituitary adenylate cyclase-activating polypeptide receptor 1
Clcf1: Cardiotrophin-like cytokine factor 1
Cntfr: Ciliary neurotrophic factor receptor
Gfap: Glial fibrillary acidic protein
Apoe: Apolipoprotein E
Kcnj10: Potassium inwardly rectifying channel subfamily J member 10
Hmgcs1: 3-hydroxy-3-methylglutaryl-CoA synthase 1
Fdft1: Farnesyl-diphosphate farnesyltransferase 1
IL-34: Interleukin 34
CSF1R: Colony stimulating factor 1 receptor
Ccl2: C-C motif chemokine ligand 2
Csf1: Colony stimulating factor 1
Th1: T helper 1
Abca1: ATP binding cassette subfamily A member 1
DPN: Diabetic painful neuropathy
Fxyd7: FXYD domain containing ion transport regulator 7
Atp1b1: ATPase Na+/K+ transporting subunit beta 1
Scn9a/SCN9A: Sodium voltage-gated channel alpha subunit 9
Scn3a: Sodium voltage-gated channel alpha subunit 3
Ptn: Pleiotrophin
Ncl: Nucleolin
Mdk: Midkine
CINP: Chemotherapy-induced neuropathic pain
Camk1d: Calcium/calmodulin-dependent protein kinase ID
Mmp14: Matrix metallopeptidase 14
Adam17: ADAM metallopeptidase domain 17
ATAC-seq: Assay for transposase-accessible chromatin using sequencing
TRPV1: Transient receptor potential vanilloid 1
NTRK3: Neurotrophic receptor tyrosine kinase 3
DCC: Deleted in colorectal carcinoma
GWAS: Genome-wide association study
Nav1.7: Voltage-gated sodium channel Nav1.7
Nav1.8: Voltage-gated sodium channel Nav1.8
SCN10A: Sodium voltage-gated channel alpha subunit 10
FDA: Food and Drug Administration
SCN11A: Sodium voltage-gated channel alpha subunit 11
EFNB2: Ephrin B2
ERO1: Endoplasmic reticulum oxidoreductin 1
SASP: Senescence-associated secretory phenotype
AAV: Adeno-associated virus
Kir2.1: Inwardly rectifying potassium channel 2.1
